# Supplementary material for: First and Second-Line Anti-Tuberculosis Drug-Resistance Patterns in Pulmonary Tuberculosis Patients in Zambia
Source: Antibiotics (Basel). 2023 Jan 12;12(1):166. doi: 10.3390/antibiotics12010166 (PMC9855139; doi:10.3390/antibiotics12010166)
Supplement: Supplementary file 1 [file antibiotics-12-00166-s001.zip › antibiotics-2124804-supplementary.pdf]

Table S1: Drug resistance profiles of the MTB isolates

Sample ID	Province	Urban/Rural	New/Previously treated	DST results	Drug Resistance profile
SM614	Southern	Urban	new	RIF mono	INH
SM602	Southern	Urban	Previously treated	INH mono + Secondline	INH, CFZ
SM511	Southern	Urban	Previously treated	MDR	INH, RIF,
SM606	Eastern	Rural	Previously treated	INH mono	INH
SM490	Southern	Urban	Previously treated	RIF mono	RIF
SM609	Southern	Urban	Previously treated	MDR	INH, RIF
SM413	Eastern	Urban	Previously treated	INH Mono	INH
SM615	Eastern	Rural	Previously treated	INH Mono	INH
SM610	Eastern	Rural	Previously treated	MDR	INH, RIF
SM386	Eastern	Rural	Previously treated	MDR + Secondline	STR, INH, RIF, EMB
SM621	Eastern	Rural	Previously treated	MDR + Secondline	STR, INH, RIF, EMB
SM695	Southern	Rural	Previously treated	INH mono	INH
SM450	Eastern	Urban	Previously treated	MDR	INH, RIF
SM815	Southern	Urban	Previously treated	RIF mono + Secondline	RR, CFZ
SM613	Eastern	Rural	Previously treated	MDR + Secondline	STR, INH, RIF, BDQ, CFZ,LEVO,MOX
SM500	Eastern	Rural	Previously treated	RIF mono	RIF
SM505	Southern	Rural	Previously treated	MDR + Secondline	STR, INH, RIF, MOX
SM423	Southern	Urban	Previously treated	STR Mono	STR
SM612	Southern	Rural	Previously treated	MDR	STR, INH, RIF
SM601	Southern	Rural	Previously treated	MDR + Secondline	INH, RIF, BDQ, CFZ
SM619	Southern	Rural	Previously treated	INH mono	INH
SM605	Southern	Urban	Previously treated	MDR	STR, INH, RIF,
SM703	Eastern	Rural	Previously treated	INH mono	INH
SM800	Eastern	Urban	Previously treated	INH mono	INH
SM801	Southern	Urban	Previously treated	INH mono + Secondline	INH, BDQ,CFZ
SM802	Eastern	Rural	Previously treated	MDR + Secondline	STR, INH, RIF, BDQ, CFZ,LEVO,MOX
SM805	Eastern	Rural	Previously treated	INH mono	INH
SM807	Southern	Urban	new	INH mono + Secondline	INH, BDQ,CFZ
SM817	Southern	Urban	Previously treated	INH mono + Secondline	INH, CFZ
SM829	Southern	Urban	Previously treated	INH mono + Secondline	INH, CFZ
SM831	Southern	Urban	new	MDR + Secondline	INH, RIF, BDQ, CFZ
SM001	Southern	Urban	New	Suceptible	Susceptible to all
SM002	Southern	Urban	New	Suceptible	Susceptible to all
SM004	Southern	Urban	New	Suceptible	Susceptible to all
SM008	Southern	Urban	New	Suceptible	Susceptible to all
SM010	Southern	Urban	New	Suceptible	Susceptible to all
SM012	Southern	Urban	New	Suceptible	Susceptible to all
SM013	Southern	Urban	New	Suceptible	Susceptible to all
SM014	Southern	Urban	New	Suceptible	Susceptible to all
SM023	Southern	Urban	New	Suceptible	Susceptible to all
SM048	Southern	Rural	New	Suceptible	Susceptible to all
SM053	Southern	Urban	New	Suceptible	Susceptible to all
SM054	Southern	Rural	New	Suceptible	Susceptible to all
SM065	Eastern	Urban	New	Suceptible	Susceptible to all

SM083	Southern	Rural	New	Suceptible	Susceptible to all
SM085	Southern	Rural	New	Suceptible	Susceptible to all
SM097	Southern	Rural	New	Suceptible	Susceptible to all
SM106	Southern	Rural	New	Suceptible	Susceptible to all
SM108	Southern	Rural	New	Suceptible	Susceptible to all
SM109	Southern	Rural	New	Suceptible	Susceptible to all
SM114	Southern	Rural	New	Suceptible	Susceptible to all
SM143	Eastern	Rural	New	Suceptible	Susceptible to all
SM257	Eastern	Rural	New	Suceptible	Susceptible to all
SM186	Eastern	Rural	New	Suceptible	Susceptible to all
SM202	Eastern	Urban	New	Suceptible	Susceptible to all
SM255	Eastern	Rural	New	Suceptible	Susceptible to all
SM256	Eastern	Urban	New	Suceptible	Susceptible to all
SM258	Eastern	Urban	New	Suceptible	Susceptible to all
SM259	Eastern	Urban	New	Suceptible	Susceptible to all
SM277	Southern	Rural	New	Suceptible	Susceptible to all
SM311	Eastern	Urban	New	Suceptible	Susceptible to all
SM312	Eastern	Urban	New	Suceptible	Susceptible to all
SM313	Eastern	Urban	New	Suceptible	Susceptible to all
SM314	Eastern	Urban	New	Suceptible	Susceptible to all
SM315	Eastern	Urban	New	Suceptible	Susceptible to all
SM316	Eastern	Urban	New	Suceptible	Susceptible to all
SM317	Eastern	Urban	New	Suceptible	Susceptible to all
SM318	Eastern	Rural	New	Suceptible	Susceptible to all
SM319	Eastern	Urban	New	Suceptible	Susceptible to all
SM320	Eastern	Rural	New	Suceptible	Susceptible to all
SM321	Eastern	Urban	New	Suceptible	Susceptible to all
SM322	Eastern	urban	New	Suceptible	Susceptible to all
SM323	Eastern	urban	New	Suceptible	Susceptible to all
SM382	Eastern	Urban	New	Suceptible	Susceptible to all
SM384	Eastern	Urban	New	Suceptible	Susceptible to all
SM385	Eastern	Urban	New	Suceptible	Susceptible to all
SM386	Eastern	Rural	New	Suceptible	Susceptible to all
SM387	Eastern	Urban	New	Suceptible	Susceptible to all
SM388	Eastern	Urban	New	Suceptible	Susceptible to all
SM389	Eastern	Urban	New	Suceptible	Susceptible to all
SM370	Eastern	Urban	New	Suceptible	Susceptible to all
SM371	Eastern	Urban	New	Suceptible	Susceptible to all
SM372	Eastern	Urban	New	Suceptible	Susceptible to all
SM373	Eastern	Urban	New	Suceptible	Susceptible to all
SM379	Eastern	Rural	New	Suceptible	Susceptible to all
SM429	Eastern	Urban	New	Suceptible	Susceptible to all
SM430	Eastern	Urban	New	Suceptible	Susceptible to all
SM431	Eastern	Urban	New	Suceptible	Susceptible to all
SM432	Eastern	Urban	New	Suceptible	Susceptible to all
SM650	Eastern	Urban	New	Suceptible	Susceptible to all
SM651	Eastern	Urban	New	Suceptible	Susceptible to all
SM653	Eastern	Urban	New	Suceptible	Susceptible to all

SM654	Eastern	Urban	New	Suceptible	Susceptible to all
SM726	Southern	Urban	New	Suceptible	Susceptible to all
SM727	Southern	Urgan	New	Suceptible	Susceptible to all
SM715	Southern	Rural	New	Suceptible	Susceptible to all
SM433	Eastern	Urban	New	Suceptible	Susceptible to all
SM434	Eastern	Urban	New	Suceptible	Susceptible to all
SM452	Eastern	Rural	New	Suceptible	Susceptible to all
SM374	Eastern	Rural	New	Suceptible	Susceptible to all
SM376	Eastern	Urban	New	Suceptible	Susceptible to all
SM377	Eastern	Rural	New	Suceptible	Susceptible to all
SM378	Eastern	Rural	New	Suceptible	Susceptible to all
SM016	Southern	Urban	New	Suceptible	Susceptible to all
SM018	Southern	Urban	New	Suceptible	Susceptible to all
SM019	Southern	Urban	New	Suceptible	Susceptible to all
SM020	Southern	Urban	New	Suceptible	Susceptible to all
SM656	Eastern	Urban	New	Suceptible	Susceptible to all
SM803	Southern	Urban	Previously treated	Suceptible	Susceptible to all
SM804	Southern	Rural	Previously treated	Suceptible	Susceptible to all
SM806	Southern	Urban	Previously treated	Suceptible	Susceptible to all
SM808	Eastern	Urban	Previously treated	Suceptible	Susceptible to all
SM809	Southern	Urban	Previously treated	Suceptible	Susceptible to all
SM810	Eastern	Urban	new	Suceptible	Susceptible to all
SM811	Southern	Urban	Previously treated	Suceptible	Susceptible to all
SM812	Southern	Urban	Previously treated	Suceptible	Susceptible to all
SM813	Eastern	Rural	Previously treated	Suceptible	Susceptible to all
SM814	Eastern	Rural	Previously treated	Suceptible	Susceptible to all
SM816	Eastern	Rural	Previously treated	Suceptible	Susceptible to all
SM818	Southern	Urban	new	Suceptible	Susceptible to all
SM819	Southern	Urban	new	Suceptible	Susceptible to all
SM820	Eastern	Rural	Previously treated	Suceptible	Susceptible to all
SM821	Eastern	Urban	Previously treated	Suceptible	Susceptible to all
SM822	Southern	Urban	new	Suceptible	Susceptible to all
SM823	Eastern	Urban	Previously treated	Suceptible	Susceptible to all
SM824	Eastern	Rural	Previously treated	Suceptible	Susceptible to all
SM827	Eastern	Rural	Previously treated	Suceptible	Susceptible to all
SM828	Eastern	Urban	Previously treated	Suceptible	Susceptible to all
SM830	Eastern	Urban	new	Suceptible	Susceptible to all
SM832	Eastern	Urban	Previously treated	Suceptible	Susceptible to all
SM825	Eastern	Rural	Previously treated	Suceptible	Susceptible to all
SM826	Eastern	Rural	Previously treated	Suceptible	Susceptible to all

STR- Streptomycin, INH- Isonaizid, RIF- Rifampicin, EMB- Ethambutol, BDQ- Bedaquiline, CFZ- Clofazimine, LEVO – Levofloxacin, MOX- Moxifloxacin, mono- mono-resistance
